# Increased femoral antetorsion results in decreased difference between the radiographic and anatomic determined Schoettle's point in MPFL reconstruction

**DOI:** 10.1002/jeo2.70376

**Published:** 2025-07-18

**Authors:** Luca Maddaloni, Thaddäus Muri, Fabio Bekcic, Lazaros Vlachopoulos, Sandro F. Fucentese, Lukas Jud

**Affiliations:** ^1^ Department of Orthopedics, Balgrist University Hospital University of Zurich Zurich Switzerland; ^2^ Research in Orthopedic Computer Science (ROCS), Balgrist University Hospital University of Zurich Zurich Switzerland

**Keywords:** ligament reconstruction, medial patellofemoral ligament (MPFL), patellar instability, patellofemoral instability, Schoettle's point

## Abstract

**Purpose:**

Medial patellofemoral ligament (MPFL) reconstruction serves as a cornerstone in surgical treatment of patellofemoral instability. An intraoperative lateral knee radiograph is used to identify the femoral insertion of the MPFL, respectively the Schoettle's point (SP). However, anatomical differences of the distal femur may impair the acquisition of the lateral knee radiograph and therefore compromise the identification of the SP.

**Methods:**

All patients who underwent MPFL‐reconstruction from January 2014 to December 2023 and with an available full radiographic dataset were included. The SP was determined both, radiographically and anatomically, using three‐dimensional (3D) surface models. The differences between the two methods were calculated and the relationship to the measured distal femoral anatomical parameters assessed using binary logistic regression.

**Results:**

Seventy knees (36 left and 34 right) in 65 patients (48 females and 17 males) were included. The mean value of the distance between the radiographic and anatomic SP was 5.1 mm ±2.5 mm, in 15 knees the distance was bigger than 7 mm. Femoral torsion was the only significant parameter in the binary logistic regression, indicating lower femoral torsion increasing the likelihood of a distance between the radiographic and anatomic SP exceeding 7 mm.

**Conclusion:**

Among all assessed distal femoral anatomical parameters, only decreased femoral torsion was associated with increased differences between the radiographic and anatomic determined SP. Hence, the intraoperative clinical control of the isometric MPFL insertion remains advisable.

**Level of Evidence:**

Level III.

**Abbreviations**
3D: three‐dimensional
aLDFA: anatomical lateral distal femoral angle
CI: confidence interval
CT: computed tomography
DFT: distal femoral torsion
FT: femoral torsion
ICC: intraclass correlation coefficient
lAPCL: lateral anterior‐posterior condyle length
LLR: long leg radiographs
mAPCL: medial anterior‐posterior condyle length
mLDFA: mechanical lateral distal femoral angle
MPFL: medial patellofemoral ligament
SA: sulcus angle
SD: standard deviation
SP: Schoettle's point
TT–TG distance: tibial tuberosity–trochlear groove distance

## INTRODUCTION

Patellofemoral instability causes 2%–3% of knee joint complaints [16]. The instability can be associated by different anatomic risk factors, including increased tibial tuberosity–trochlear groove (TT–TG) distance, patella alta, increased femoral torsion or trochlear dysplasia [1, 7, 8, 11, 21]. In case of surgical treatment of patellofemoral instability, a thorough assessment of the anatomical parameters is of paramount. Beside correcting the pathologic anatomical parameters, the reconstruction of the medial patellofemoral ligament (MPFL) is frequently performed [6].

Different techniques have been described to identify the ideal femoral insertion of the MPFL intraoperatively. Nowadays, the most common accepted technique is the use of the Schoettle's point (SP) [19]. The correct identification of the SP requires a lateral knee radiograph with perfectly overlaid posterior femoral condyles [3, 31]. The correct identification of the femoral MPFL insertion is essential, as malpositioning is a leading cause of clinical failure [24, 27]. However, anatomical variations of the distal femur may complicate the acquisition of such a radiograph, necessitating more intense knee manipulation to achieve the desired overlay. This manipulation may lead to a wrong projection of the radiographically determined SP, differing from the anatomically determined SP. We hypothesised that alterations of the distal femoral anatomical parameters impair the radiological identification of the femoral insertion of the MPFL. Therefore, the aim of this study was to examine the relationship between different distal femoral anatomical parameters and the difference between the radiographically and anatomically determined SP.

## MATERIAL AND METHODS

### Patient selection

All patients who underwent MPFL‐reconstruction from January 2014 to December 2023 at our institution were included. Further inclusion criterion was the availability of a full radiographic data set, which includes conventional knee radiographs, long leg radiographs (LLR) and a preoperative computed tomography (CT) of the affected lower extremity, including the hip and knee joint. Exclusion criterions were osteoarthrosis, immature skeleton, previous fractures or bony procedures on the same knee. This study was approved by the local ethical committee (Zurich Cantonal Ethics Commission, BASEC‐Nr. 2023‐01376).

### Radiological assessment

**Figure 1:**
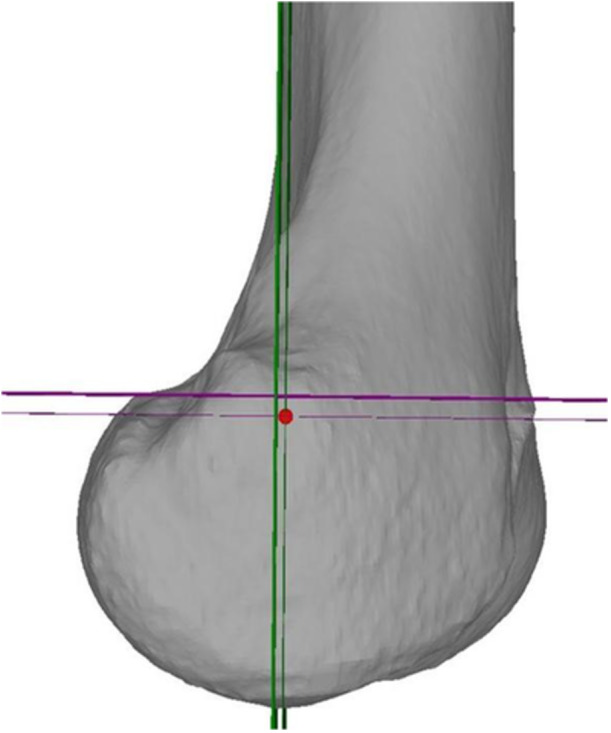
Determination of the radiographic Schoettle's point (red) according to the posterior femoral cortical line (green, thick) and 1.3 mm anterior to the posterior femoral cortical line (green, thin) as well as the perpendicular epicondylar line (purple, thick) and 2.5 mm distal to the perpendicular epicondylar line (purple, thin).

**Figure 2:**
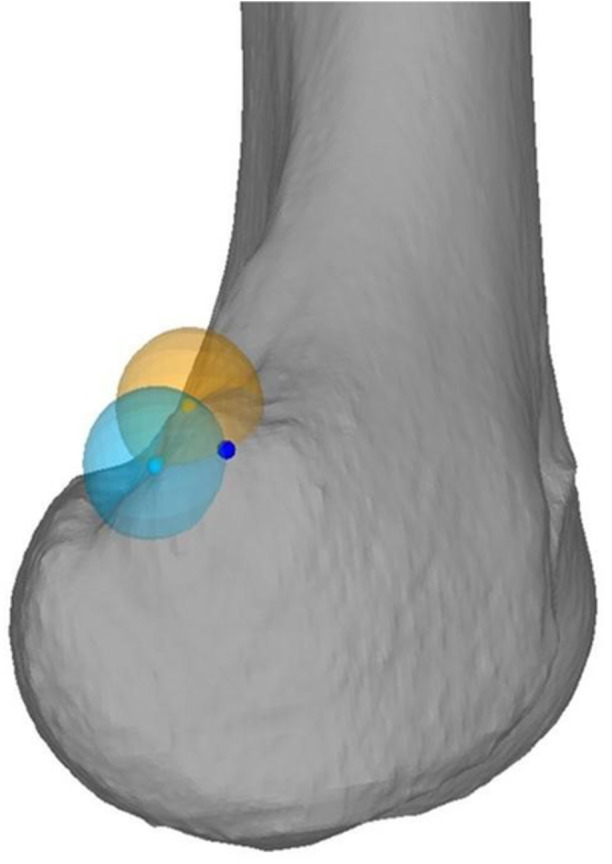
Determination of the anatomical Schoettle's point (SP) (dark blue) 8 mm distal (transparent orange ball with 8 mm radius) to the apex of the tuberculum adducorium (orange) and 8 mm anterior (transparent light blue ball with 8 mm radius) to the posterior edge (light blue) The anatomical SP (dark blue) represents the intersection of the transparent light blue and the transparent orange ball.

CT scans were segmented using MIMICS (Materialize, Leuven, Belgium) to generate three‐dimensional (3D) triangular surface models of the femur. The 3D models were then transferred to the in‐house developed surgical planning software CASPA (Balgrist, CARD, Zurich, Switzerland), where the identification of the radiographic and anatomic SP was performed. Therefore, the distal femur was aligned to an orientation with a perfect overlay of the posterior femoral condyles, corresponding to a lateral radiograph of the knee. Based on this orientation, the radiographic SP was determined according to the description of Schoettle et al. [19] (Figure 1). The anatomical SP was determined using the tuberculum adductorium and the posterior edge (i.e., the edge of the posteromedial cortex between the medial condyle and femoral shaft), according to the technique described by Wang et al. [28] (Figure 2).

**Figure 3:**
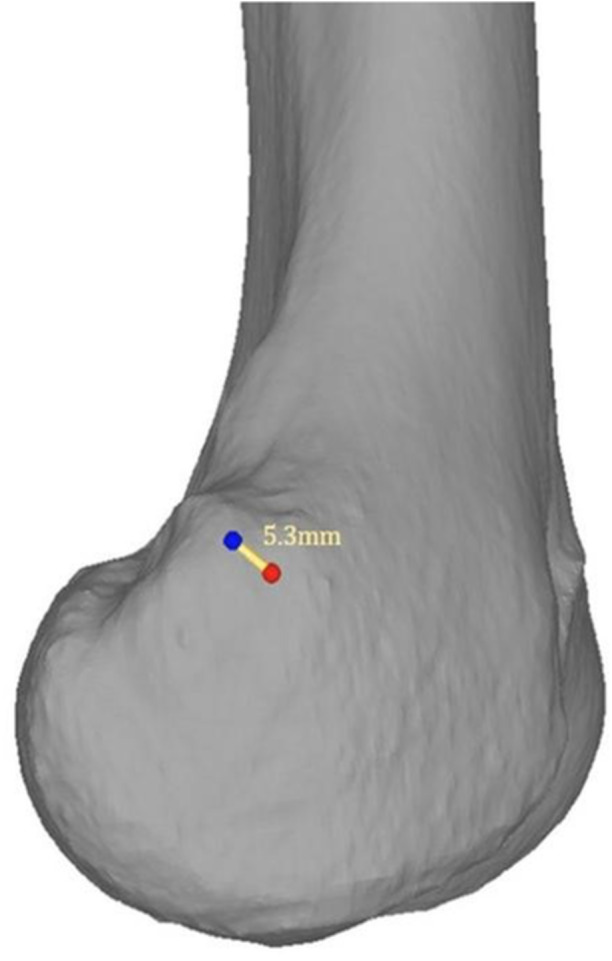
Distance between the radiographic (red) and anatomic (blue) Schoettle's point (SP) visualised in yellow. In this case, the distance measured 5.3 mm.

According to Servien et al. [20], a femoral MPFL tunnel is considered malpositioned if it differs >7 mm from the correct SP. Therefore, the distance between the radiographic and anatomic SP was calculated (Figure 3) and a difference of >7 mm was considered as insufficient. Following, patients were grouped in patients with a sufficient identification of the radiographic SP (i.e., difference between radiographic and anatomic SP ≤ 7 mm) and patients with an insufficient identification of the radiographic SP (i.e., difference between radiographic and anatomic SP > 7 mm).

The following distal femoral anatomical parameters were evaluated: to determine the distal femoral torsion (DFT), the angle between the transepicondylar axis and the posterior bicondylar axis was measured according to Rougereau et al. [17]. The medial and lateral anterior‐posterior condyle length (mAPCL respectively lAPCL) were measured using the greatest distance of the perpendicular line to the posterior bicondylar axis travelling through the respective condyle [17]. The anatomical and mechanical lateral distal femoral angles (aLDFA respectively mLDFA) were measured in LLR according to Paley et al. [15]. The femoral torsion was measured according to the method described by Waidelich et al. [26]. The sulcus angle was measured according to the technique described by Davies et al. [5].

All measurements were performed by two independent readers (LM, TM; orthopaedists) for inter‐rater reliability.

### Statistical analysis

Sociodemographic and clinical characteristics of patients were determined using descriptive statistics. Continuous variables are reported as mean and standard deviation (SD). Normality of distribution was tested using the Shapiro–Wilk test. Accordingly, univariate analysis was performed using unpaired t‐test or Mann–Whitney *U* test to assess differences of distal femoral anatomical parameters and height between two groups. Multivariate analysis was performed for significant parameters using binary logistic regression. Model fit was assessed using Hosmer–Lemeshow test. Inter‐rater reliability was calculated with the intraclass correlation coefficient (ICC). ICCs were interpreted according to Landis and Koch [14]. Post hoc power analysis revealed a power of 95% to detect a significant difference in FT between the groups at a α level of 0.05. Statistical significance was set at *p* < 0.05. Mean values of both readers were used for analysis. All statistical analyses were performed in SPSS for Windows (Version 29.0, SPSS Inc., Chicago, Illinois).

## RESULTS

Seventy knees (36 left and 34 right) in 65 patients (48 females and 17 males) were included. The mean age was 22.0 ± 6.7 years, mean height was 170.8 ± 9.3 cm, and mean body mass index (BMI) was 24.3 ± 4.8 kg/m^2^. The mean difference between the radiographic and anatomic SP was 5.1 mm ± 2.5 mm (range: 0.6–11.8 mm). Fifteen knees showed an insufficient identification of the radiographic SP with a difference between the radiographic and the anatomic SP > 7 mm, whereas 55 knees showed a sufficient identification of the radiographic SP with a difference ≤ 7 mm. An overview of the measured radiographic parameters is given in Table 1. The univariate analysis showed significant difference between two groups for lAPCL (*p* = 0.024) and femoral torsion (*p* < 0.001). Furthermore, height was significantly different between two groups in the univariate analysis (*p* = 0.027). The following binary logistic regression revealed significance only for femoral torsion (*p* < 0.001). It showed a decreased odd in increased femoral torsion for having a difference between the radiographic and anatomic SP of >7 mm (odds ratio, 0.82; *p* < 0.001).

**Table 1:** Radiographic measurements of the distal femoral anatomical parameters.

	All patients (*n* = 70)	Insufficient group (*n* = 15)	Sufficient group (*n* = 55)	*p* value
DFT (°)	6.1 ± 1.9 (range: 2.1–9.3)	6.6 ± 1.9 (range: 4.3–8.8)	6.0 ± 2.0 (range: 2.1–9.3)	n.s.
lAPCL (mm)	60.8 ± 4.4 (range: 48.0–71.0)	62.5 ± 4.3 (range: 50.4–69.0)	60.3 ± 4.3 (range: 48.0–71.0)	0.024
mAPCL (mm)	57.8 ± 4.3 (range: 45.6–67.5)	59.4 ± 5.0 (range: 45.6–66.6)	57.3 ± 4.0 (range: 49.2–67.5)	n.s.
mLDFA (°)	86.7 ± 2.7 (range: 80.7–92.8)	86.9 ± 3.2 (range:81.2–91.2)	86.6 ± 2.5 (range: 80.7–92.8)	n.s.
aLDFA (°)	80.6 ± 2.9 (range: 74.7–86.1)	81.0 ± 3.4 (range: 74.8–85.6)	80.5 ± 2.7 (range: 74.7–86.1)	n.s.
FT (°)	25.9 ± 10.9 (range: 3.3–51.4)	17.5 ± 9.3(range: 3.3–30.8)	28.2 ± 10.3(range: 4.0–51.4)	<0.001
SA (°)	142.0 ± 9.3 (range: 120.2–165.7)	142.9 ± 1.9 (range: 127.1–165.7)	141.7 ± 8.6 (range: 120.2–159.5)	n.s.

Abbreviations: aLDFA, anatomical lateral distal femur angle; DFT, distal femoral torsion; FT, femoral torsion; lAPCL, lateral anterior‐posterior condyle length; mAPCL, medial anterior‐posterior condyle length; mLDFA, mechanical lateral distal femur angle; SA, sulcus angle.

The ICC between the two readers for measuring the anatomical parameters showed 'almost perfect agreement' for the lAPCL [0.95 (95%CI: 0.93–0.97)], mLDFA [0.95 (95%CI: 0.93–0.97)], aLDFA [0.95 (95%CI: 0.92–0.97)], femoral torsion [0.98 (95%CI: 0.96–0.98)] and the sulcus angle [0.85 (95%CI: 0.76–0.91)]. ICC for DFT [0.74 (95%CI: 0.58–0.84)] and the distance between the radiographic and anatomic SP [0.79 (95%CI: 0.56–0.90)] showed 'substantial agreement'. 'Moderate agreement' was achieved for the mAPCL [0.58 (95%CI: −0.18 to 0.85)].

## DISCUSSION

The most important finding of this study is that of the assessed distal femoral anatomical parameters, only a decreased femoral torsion showed an increased odd for having a discrepancy between the radiographic and anatomic determined SP of >7 mm, considered as an insufficient femoral insertion of the MPFL. Accordingly, these findings confirm our hypothesis. Hence, the identification of the radiographic SP seems safe regarding most distal femoral anatomical parameters.

MPFL reconstruction serves as a cornerstone in surgical treatment of patients with patellofemoral instability and is performed in most cases [6]. The correct identification of the femoral insertion of the MPFL is mandatory, as malpositioning of the MPFL is a leading cause of clinical failure [2, 22, 24, 27]. Various techniques have been described to identify the ideal femoral insertion of the MPFL, including anatomic and radiographic techniques [4, 9, 25, 29]. The anatomic technique is based on palpation of the medial epicondyle and the tuberculum adductorium [4]. Regarding the radiographic method, the use of the SP is probably the most used technique nowadays [12, 13, 19, 22, 30]. The identification of the SP is performed by using intraoperative fluoroscopy, respectively by acquisition of an intraoperative lateral knee radiograph. However, there have been concerns using the radiographic technique due to inaccuracy of the identification of the femoral MPFL insertion, especially in patients with trochlear dysplasia [10, 18]. Considering the finding, that different anatomy of the distal femur in terms of trochlear dysplasia leads to inaccurate identification of the SP, this study aimed to investigate if also other distal femoral anatomical parameters impair the identification of the SP. In this study, considering different common distal femoral anatomical parameters in patients which have been subject to a MPFL reconstruction at our clinic, the sulcus angle, in terms of a trochlear dysplasia, did not show an increased risk for an inaccurate identification of the SP, in contrast to the previous literature [10, 18, 23, 27]. The observed difference in lAPCL between the two groups did not remain significant in the binary logistic regression. Consequently, it is likely a surrogate marker rather than an independent predictor. A decreased femoral torsion showed to be the only distal femoral anatomical parameter with an increased odd for inaccurate identification of the radiographic SP in this study. This is probably explained by the different rotation of the distal femur required for the perfect overlay of the posterior femoral condyles. However, a definitive explanation for this finding cannot be given at this point. Nevertheless, the radiographic SP appears to be a reliable technique for identifying the femoral MPFL insertion in regards of different anatomy of the distal femur, but the additional intraoperative clinical control of the isometric insertion of the MPFL seems advisable.

This study should be interpreted in lights of its potential limitations. First, absolute values were used to determine the anatomical SP (i.e., 7 mm). Due to this, patient's height could be a cofounder. However, even though height was significant in the univariate analysis between two groups, it showed no significant difference in the binary logistic regression. This suggests that height may act as a surrogate marker rather than an independent predictor. Second limitation is the use of a computer simulation approach for calculation of the difference between the radiographic and anatomic SP. Cadaver experiments could be performed for a more comprehensive analysis. However, efforts and costs for cadaver experiments are very high and computer simulation approaches are established and cost‐effective alternatives.

## CONCLUSION

Among all assessed distal femoral anatomical parameters, only decreased femoral torsion was associated with increased differences between the radiographic and anatomic determined SP. Hence, the intraoperative clinical control of the isometric MPFL insertion remains advisable.

## AUTHOR CONTRIBUTIONS

All authors have made substantial contributions to all of the following: (1) the conception and design of the study, or acquisition of data, or analysis and interpretation of data, (2) drafting the article or revising it critically for important intellectual content, (3) final approval of the version to be submitted.

## CONFLICT OF INTEREST STATEMENT

The authors declare no conflicts of interest.

## ETHICS STATEMENT

The local ethical committee approved this retrospective study (Zurich Cantonal Ethics Commission, BASEC‐Nr. 2023‐01376). Informed consent was obtained from all study participants.

## Data Availability

The data that support the findings of this study are available on request from the corresponding author. The data are not publicly available due to privacy or ethical restrictions.
